# The Influence of the Upholstery Textiles Structure on Their Functional Properties

**DOI:** 10.3390/ma18225143

**Published:** 2025-11-12

**Authors:** Justyna Pinkos, Adam K. Puszkarz, Jacek Rutkowski, Martyna Borowińska

**Affiliations:** 1 Institute of Architecture of Textiles, Faculty of Textiles and Design, Lodz University of Technology, 116 Żeromskiego Street, 90-924 Lodz, Poland; martyna.borowinska@gmail.com; 2 Textile Institute, Faculty of Textiles and Design, Lodz University of Technology, 116 Żeromskiego Street, 90-924 Lodz, Poland

**Keywords:** upholstery textiles, woven fabric, knitted fabric, air permeability, tensile strength, abrasion, pilling, flammability, micro-CT

## Abstract

This article evaluates selected performance parameters of commercial upholstery textiles that impact on user comfort and safety. The study included four commercial multilayer textiles: RIBCORD bright, RIBCORD dark, OREO, and ILIAS, each with varying morphology (thickness, number of layers, porosity, and raw material composition). Statistical analysis of the textile microstructure was conducted using high-resolution micro-computed tomography (micro-CT). The performance parameters of the textiles were determined based on tests of air permeability, tensile strength, abrasion and pilling resistance, and flammability. The significantly different results obtained for all performance parameters of the tested textiles are justified by the results of the microstructure analysis. A clear correlation was observed between air permeability and the porosity of the inner layer of all tested textiles. Tensile strength tests revealed significant mechanical anisotropy in all textiles. Abrasion and pilling resistance tests demonstrated very good properties for RIBCORD bright, RIBCORD dark, and OREO. Flammability tests have shown that OREO is a flame-retardant textile material.

## 1. Introduction

Ensuring fire safety in public utility buildings is one of the key challenges associated with the protection of life and property. A wide variety of materials in the form of fabrics, artificial leather, foils, foams, and composites are used for furnishing interiors with furniture and decorative elements. All commonly used materials, when exposed even to low-energy ignition sources, ignite and subsequently spread flame while releasing smoke and toxic combustion products [1,2,3]. For this reason, products constituting the interior furnishings of public utility buildings, such as upholstered furniture, floor coverings, curtains, and draperies, are subjected to detailed testing. Safety considerations exclude the use of materials that release enormous amounts of smoke and toxic volatile substances during a fire. Reducing the flammability of polymer materials to ensure human safety and protect property has thus become one of the priority safety requirements [4,5,6]. During flammability testing, parameters are determined to allow the proper classification of the material under examination [7,8,9]. Based on these results, a given material is either accepted or rejected for a specific application in a defined location [10,11]. Not all users of different types of rooms in public utility buildings are aware of the degree of fire risk, which means they do not always exercise the necessary caution that determines their safety. Fires occurring inside buildings constitute a significant portion of all fires. According to statistics from the Headquarters of the State Fire Service, in 2024, there were nearly 50,000 fires in Poland, of which nearly 30,000 were in residential buildings and about 3000 in public buildings [12].

Upholstered furniture includes a variety of interior furnishings such as chairs, armchairs, sofas, couches, and ottomans, which combine functionality and comfort. Upholstered furniture is standard equipment in many administrative buildings, offices, healthcare facilities, as well as commercial and service facilities. Their common feature is a similar construction and the use of specific materials. They typically consist of a wooden or metal frame, soft filling, and upholstery. The filling is most often flammable polyurethane foam, while the upholstery is made of furniture fabrics produced from common fibers [13,14]. It is worth noting that these materials are, in many cases, not resistant to fire, which can pose a serious hazard in contact with fire. The selection of materials for upholstered furniture, ensuring they do not constitute a fire risk, is therefore of fundamental importance. Such furniture cannot be manufactured using random, arbitrary materials. Most commonly available materials used in furniture production have low ignition resistance, which means that these products may contribute to the initiation of a fire within a building. To prevent such situations, greater attention must be paid to the properties of materials, including flammability, smoke generation, toxicity of thermal decomposition and combustion products, heat release rate, and flame spread capability [15,16,17]. The development of a fire depends not only on the properties of the materials from which interior furnishings are made, but also on environmental conditions, such as temperature and airflow in the room. Foams used in upholstered furniture production exhibit high smoke generation and toxic gas emission during fire. Even a small amount of polyurethane foam is sufficient, during thermal decomposition and combustion, to exceed the permissible threshold concentrations of toxic gases such as carbon monoxide and hydrogen cyanide [18]. Upholstery fabrics also release hazardous substances during burning. Fire statistics show that 60–80% of fire fatalities result from smoke toxicity rather than direct exposure to flames [19,20,21].

In the United States, between 2009 and 2011, an average of about 5000 fires per year were recorded where the primary cause was upholstered furniture. These events resulted in an average of 410 deaths annually, and about 730 people sustained injuries [22,23]. The situation in Poland is also concerning. In recent years, the average number of fatalities from fires has been about 500 people annually. In 2024 alone, more than 27,400 residential building fires were recorded, in which more than 1900 people were injured and 269 lost their lives [24]. Available statistical data clearly indicate the impact of insufficient fire safety regulations on the number of fatalities. Incidents related to the flammability of upholstered furniture occur in a wide range of locations and conditions—from residential buildings, through industrial facilities, to vehicle interiors. The broad spectrum of potential ignition sites highlights the need for continued research on the flammability of materials used in furniture production and the implementation of more effective legislative and technological solutions to reduce fire risk.

It should be noted that between 2020 and 2022, global furniture sales grew at an average annual rate of 5.8%, and in 2023, increased by 3.6%, reaching a value of nearly EUR 683 billion. In 2023, North America was recognized as the largest furniture sales market, valued at EUR 261 billion, accounting for 38.2% of global sales. China was the world leader in furniture exports, with a market share of 39.5% in 2023. The United States was the largest importer in 2023, with a 26% share of global imports. Meanwhile, Poland ranked as the world’s third-largest furniture exporter, with a market share of 5.8%, and the tenth-largest importer, with a 1.9% share [25]. It should be emphasized that the growing trend in furniture sales generates the need for fire safety regulations for upholstered furniture. In the European Union, there are no unified fire protection regulations. However, the flammability testing method is standardized and defined in European standards [26].

This article presents the results of tests on commercially available upholstery textiles (three woven fabrics and a knitted fabric) used in the production of couches, sofas, and armchairs. The main aim of the study was to assess the effect of the structure of the tested textiles on their functional parameters. The layered structure of the textiles (with different spatial geometry, raw material composition, and surface mass) was examined using high-resolution X-ray microtomography (micro-CT). This technique enabled the calculation of basic textile parameters (total thickness of woven and knitted fabrics, as well as the thickness of their individual layers). Furthermore, the microstructure of the textiles was characterized using statistical analysis and distributions: fiber/yarn diameter and pore size. The total porosity, yarn porosity, and the porosity of individual layers were determined for each textile material.

The tests on the functional parameters of the textiles were related to comfort and safety of use and included air permeability, tensile strength, abrasion and pilling resistance, and flammability assessment.

## 2. Materials and Methods

### 2.1. Materials

**Figure 1:**
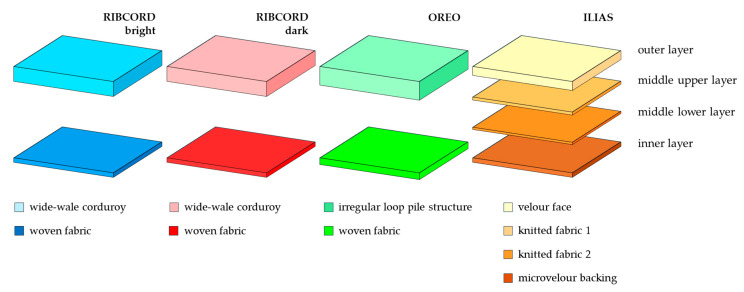
Scheme of the layered structure of the tested textiles.

The subject of the research was 4 textile products with potential use as upholstery materials: three woven fabrics: RIBCORD bright, dark RIBCORD dark, OREO, and knitted fabric: ILIAS (Glormeb Group, Warsaw, Poland). The adjectives “bright” and “dark” refer to the actual shades of the two RIBCORD fabrics. In reality, RIBCORD bright is white, while RIBORD dark is gray. To enhance the clarity of the data presented in the article, the authors assigned artificial contrasting colors (blue, red, green, and orange) to the tested textiles. These colors were used in all diagrams, photos, and graphs. All three woven fabrics had a two-layer structure, while the ILIAS knitted fabric consisted of four layers. Scheme of the layered structure of the tested textiles is presented in Figure 1.

Table 1, presenting the parameters of the tested textiles, shows that they differ in terms of raw material composition, thickness (determined according to EN ISO 5084:1999 [27]), mass per unit area (determined according to EN ISO 12127:2000 [28]), and weave. The ILIAS knit fabric consists of two knitted fabrics differing in thickness and weave, which form its two middle layers (top and bottom). A common feature of all textiles is that their outer layer is by far the thickest of all the others. This layer is a fibrous covering.

**Figure 2:**
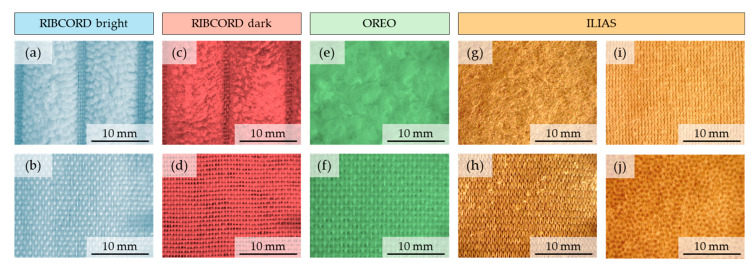
Optical microscopy images of tested textiles. RIBCORD bright: (**a**) outer layer, (**b**) inner layer; RIBCORD dark: (**c**) outer layer, (**d**) inner layer; OREO: (**e**) outer layer, (**f**) inner layer; ILIAS: (**g**) outer layer, (**h**) middle upper layer, (**i**) middle lower layer, (**j**) inner layer.

Figure 2 shows images of the surfaces of all layers constituting the 4 tested textiles, taken using optical microscopy (Delta Optical Smart 5MP PRO, Delta Optical, Warsaw, Poland) assisted by the software: Delta Optical Smart Analysis Pro 1.0.0). To visualize the surface of each ILIAS layer, they were separated from each other. Based on images taken with the same magnification, one can observe a similar structure of both layers of RIBCORD bright and RIBCORD dark woven fabrics. Unlike the other two textiles, in RIBCORD bright and RIBCORD dark, the outer layer is not continuous and forms parallel stripes (wide-wale corduroy) between which the inner layer (fabric) is transparent. Furthermore, a clear difference in structure can be seen between the woven fabrics forming the inner layer in OREO and the RIBCORD bright and RIBCORD dark. The images also show the difference in the structure of the two fabrics forming the middle layers in ILIAS, as well as the different structures of its outer and inner layers.

### 2.2. Methods

#### 2.2.1. Structure Analysis of Textiles

High-resolution X-ray microtomography, micro-CT (SkyScan 1272 Bruker, Kontich, Belgium) was used to determine the structure of the tested textiles scanned under the following conditions: X-ray source voltage of 50 kV, X-ray source current of 200 µA, image resolution 4032 × 2688, voxel size of 6 µm, rotation step of 0.2° without filter. Based on the microtomography, statistical distributions of the fiber diameters of the textiles were calculated, as well as the distribution of pores—air-filled spaces between the fibers. Furthermore, the total porosity of the textiles, yarn porosity, and the porosity of their individual layers were determined. The main goal of tomography, in addition to understanding the microstructure of the textiles, was to determine its impact on the functional parameters, the values of which were determined in this article. Statistical distributions and porosity results of the tested textiles were obtained based on tomography of 10 randomly selected samples of each material. Micro-CT analysis was successfully used by the authors of this article to investigate the effect of structure on the performance parameters of fabrics with PVD Parylene C coatings [29,30].

#### 2.2.2. Air Permeability of Textiles

The effect of different textile structures on their air permeability was determined using the method described in the EN ISO 9237:1998 [31], using an air permeability tester (FX 3300, Textest Instruments, Schwerzenbach, Switzerland). Textile tests were conducted under normal climate conditions in accordance with the EN ISO 139:2006 [32], for which the air temperature is 20 °C ± 2 °C and relative air humidity 65% ± 4%. The textiles were previously conditioned for 24 h under these conditions.

#### 2.2.3. Tensile Strength: Maximum Force and Elongation at Maximum Force of Textiles

In the next stage of the study, the maximum force and the elongation at maximum force were determined in accordance with the EN ISO 13934-1 [33]. To determine the tensile strength of textile products, a Hounsfield H10KeS universal testing machine (Hounsfield, USA) was used. Samples for measurement were prepared by cutting 5 strips in the warp direction and 5 strips in the weft direction from the tested material, each with dimensions of 50 × 200 mm. These prepared samples were placed in the machine grips, and tensile strength testing was carried out at a speed of 100 mm·min^−1^. After the strip broke, the maximum force value and the elongation at maximum force were recorded.

#### 2.2.4. Abrasion Resistance of Textile Products

**Figure 3:**
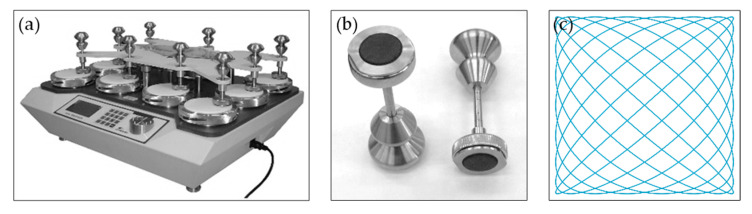
(**a**) Martindale apparatus, (**b**) sample holder, (**c**) Lissajous Figure.

Abrasion resistance testing is one of the most important parameters required when evaluating textile furniture products, and it has a significant impact on determining their quality. The studies were conducted according to a standardized test procedure according to the International Standard EN ISO 12947-2 [34]. A circular sample fixed in a sample holder, under a certain load, performing a translational movement along the paths of the Lissajous figure, is abrased by an abrasive medium (standardized flat product). The sample holder also rotates freely around its axis, perpendicular to the sample surface. The abrasion resistance rating of a flat textile product is determined on the basis of the inspection interval to the breakdown of the samples. The inspection interval is determined by the sample breakdown. The number of rubs is recorded at which the sample breakdown is not yet observed. M235 Martindale apparatus made in SDL ATLAS (Charlotte, NC 28217-2956, USA) for testing the resistance of flat fiber products for abrasion and pilling, the sample holder and Lissajous Figure are shown in Figure 3.

Equipment and auxiliary materials: sample cutter (ϕ 38 mm and ϕ 140 mm), magnifier or microscope (8×), abradant (standardized wool fabric according to EN ISO 12947-1:2000), felt (standardized woven wool felt according to EN ISO 12947-1:2000), standardized polyurethane foam (according to EN ISO 12947-1:2000), weight 795 g and 595 g (nominal pressure 12 kPa and 9 kPa), weight of 2.5 ±0.5 kg and a diameter of 120 ± 10 mm [35].

The tested samples must be acclimatized for a period of not less than 18 h, under normal climate conditions, relative humidity 65 ± 4%, air temperature 20 ± 2 °C.

#### 2.2.5. Pilling Resistant Testing of Flat Textile Products

The tests were conducted according to the standard EN ISO 12945-2:2021-04 [36].

The tests are carried out on the M235 MARTINDALE device made in SDL ATLAS (Charlotte, NC 28217-2956, USA). The apparatus is designed to determine the resistance of flat textile products to abrasion and pilling. The samples are rubbed against the abrasive material with low pressure, and the level of pilling or abrasion is compared to the standard values.

Pilling is formed when neps appear on the surface of the material and the surface pills during wearing. Such deterioration of surface quality is usually undesirable, and the degree of acceptance by users of a given level of pilling depends on the type of clothing and the purpose of the material.

In general, the level of pilling depends on the following factors:

▪ Neps-forming properties of the fibers.
▪ Presence of puffed fibers on the surface.
▪ Surface damage to the fibers.

The level of these processes depends on the fiber, type of yarn, and material construction. Examples of extreme situations can be found in materials containing durable fibers and materials containing weak fibers. The consequence of the strength of the fiber is the speed of formation of neps, which exceeds the level of wear. This results in an increase in pillng as consumption increases. With weak fibers, the speed of neps formation goes hand in hand with material consumption.

#### 2.2.6. Flammability Assessment—Ignition Source Match Flame Equivalent of Textiles

**Figure 4:**
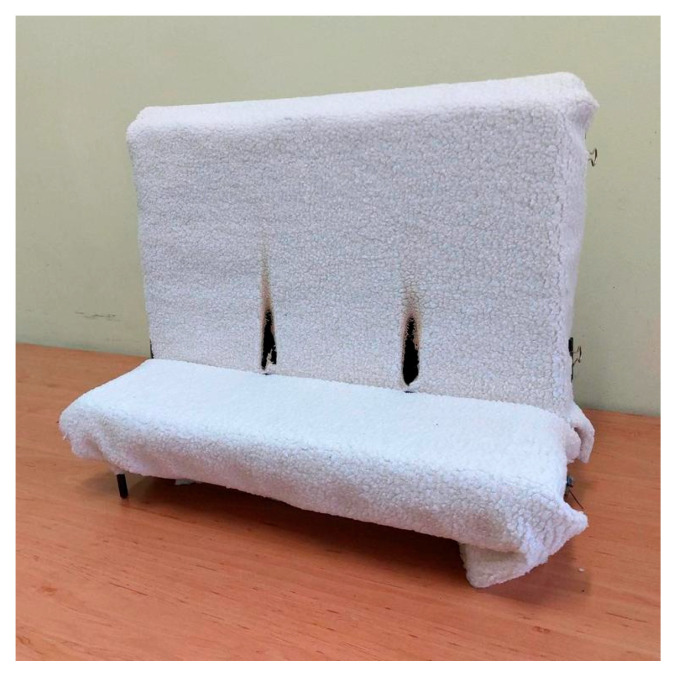
Example upholstery system after flammability testing of OREO.

A key test of upholstery systems concerned the flammability assessment—ignition source equivalent to a match flame—in accordance with EN 1021-2:2014 [26]. The upholstery system, formed from upholstery fabric samples with dimensions of 800 mm × 600 mm and two PU foam samples with dimensions 450 mm × 350 mm × 75 mm and 450 mm × 150 mm × 75 mm, was placed on the flammability testing apparatus (Figure 4) In the tested system flame retardant polyurethane foam with a thickness of 50 mm and a specific mass of 25 kg·m^−3^ was used as a filling.

The tested upholstery system is exposed to an ignition source in the form of a 35 mm high flame, generated by a special propane-fueled burner. The flame is regarded as equivalent to a match flame. The burner is positioned along the junction of the vertical (backrest) and horizontal (seat) parts of the specimen. The exposure time of the system to the flame is 15 s. At the moment the burner flame is applied to the tested specimen, a timing device (stopwatch) is activated. The course of the test is then observed, and, in accordance with the requirements of the standard, phenomena such as ignition of the specimen, glowing, and smoldering are recorded. After the flame is removed from the tested specimen, the system is further observed for the occurrence of the aforementioned phenomena. After 120 s, if no visible signs of burning, smoldering, or glowing of the upholstery system are present, the system is dismantled to check for possible non-visible internal effects (smoldering or glowing) within the filling material. Based on this examination, the final evaluation and classification of the tested upholstery system is made. If no progressive smoldering or glowing of the filling is found, the result is recorded as “no ignition,” and the tested system is deemed to meet the requirements of the standard [26].

In Table 2, a summary of the research methods used was presented.

## 3. Results and Discussion

### 3.1. Structure Analysis of Textiles

**Figure 5:**
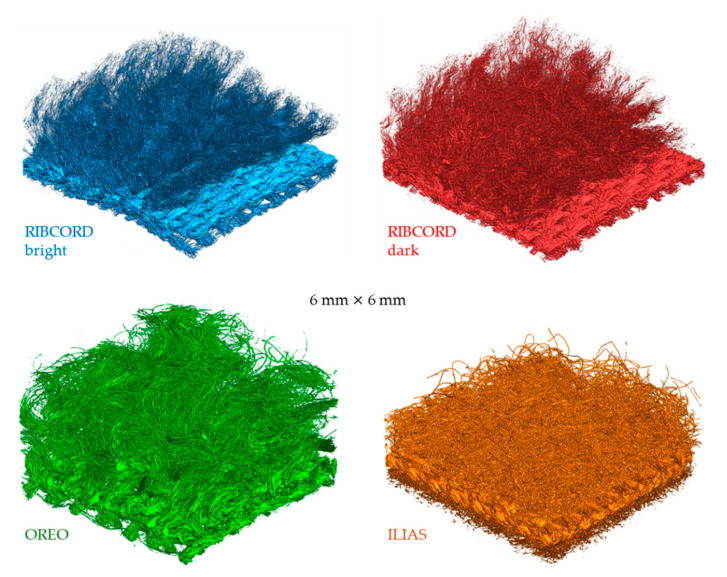
Three-dimensional micro-CT visualizations of tested textiles.

Figure 5 shows three-dimensional visualizations of textiles obtained using micro-CT. The surface of all textiles in the visualizations was limited to a square fragment measuring 6 mm × 6 mm.

The visualizations can be used to identify the layers constituting the four tested textiles. The visualizations show a clear difference in thickness between the outer and inner layers in three fabrics (RIBCORD bright, RIBCORD dark, and OREO). In the case of ILIAS, the two knitted fabrics constituting its two middle layers are tightly bonded and, together with the inner layer (microvelour backing), form part of a textile product with a thickness similar to that of the inner layers of the other three woven fabrics. Furthermore, it can be observed that the outer layer in each of the four tested textiles is composed of separate fibers that are not spun into yarns, which is why it is characterized by greater fluffiness and transparency compared to the inner layer.

**Figure 6:**
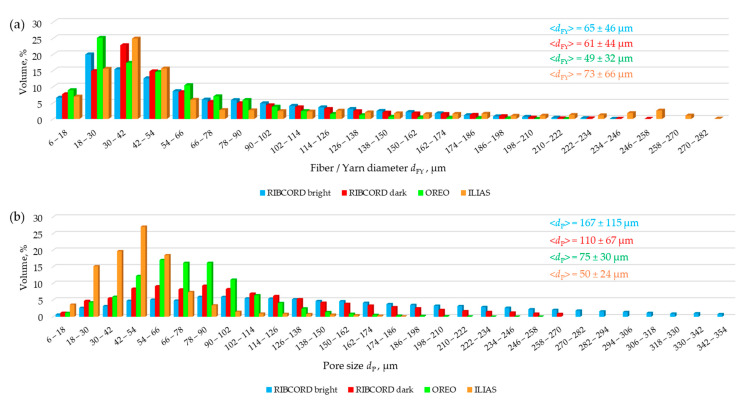
(**a**) Fiber diameter distribution in the tested textiles; (**b**) Pore size distribution in the tested textiles.

Figure 6a shows the fiber/yarn diameter distributions *d*_FY_ in the tested textiles. In all tested textiles, individual fibers occur separately, essentially only in the outer layer. In the inner layer, they are formed into yarns, creating woven fabrics (RIBCORD bright, RIBCORD dark, and OREO) and knitted fabrics (ILIAS). The fibers in the yarn are parallel, twisted, and adjacent to each other. The spatial resolution of the micro-CT (voxel size: 6 µm) makes it impossible to identify adjacent individual fibers, treating them as objects of larger diameter. Therefore, in the presented distributions, the intervals with higher diameters correspond to the diameters of objects formed from fibers forming yarns in the woven and knitted fabrics of the tested textiles.

The distributions for RIBCORD bright and RIBCORD dark have very similar shapes, indicating a similar geometric structure of these textiles. The fibers in these two fabrics have a diameter *d*_FY_ ranging from 6 µm to 258 µm, and their average diameter is very similar (<*d*_FY_> = 65 µm for RIBCORD bright, <*d*_FY_> = 61 µm for RIBCORD dark). The *d*_FY_ distributions in the other two textiles clearly differ from those mentioned above. In the case of OREO, the distribution shape is clearly narrower, with fibers reaching diameters ranging from 4 µm to 222 µm (<*d*_FY_> = 49 µm), while in the case of ILIAS, the distribution shape is more flattened, with fibers ranging in diameter from 4 µm to 282 µm (<*d*_FY_> = 73 µm).

Figure 6b shows the pore size *d*_P_ (voids between fibers) distributions in the tested textiles. As with the pore diameter distributions, the pore diameters for RIBCORD bright and RIBCORD dark have a similar shape, indicating a similar geometric structure. Pores in these two fabrics range in size from 6 µm to 270 µm (RIBCORD bright) and from 6 µm to 354 µm, with an average size: <*d*_P_> = 167 µm for RIBCORD bright, <*d*_P_> = 110 µm for RIBCORD dark. The pore size distributions in the remaining two textiles are clearly narrower, with pores ranging from 6 µm to 258 µm (<*d*_P_> = 75 µm) in OREO, while in ILIAS, pores range in size from 6 µm to 186 µm (<*d*_P_> = 50 µm). It is important to understand the pore diameter in the above distributions. The wide range of pore sizes in the tested textiles results from the diverse structure of each of the four materials. Smaller pore sizes correspond to smaller spaces between fibers within the yarn in woven fabrics (RIBCORD bright, RIBCORD dark, OREO) and knitted fabrics (ILIAS), while larger spaces between fibers occur in the outer layers of all four textiles.

**Figure 7:**
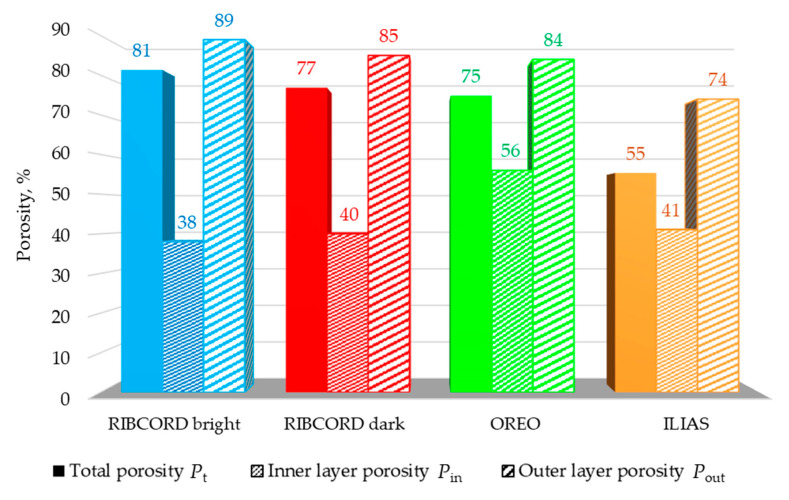
Textiles porosity.

Because the woven and knitted fabrics comprising the layers of the tested textiles have only open pores, porosity is a parameter that correlates with air permeability. Air permeability is one of the important performance parameters of upholstery textiles, influencing heat transfer through the textile and moisture wicking. The layer that determines the air permeability of textiles is usually the least porous layer. High-resolution computed microtomography enabled the examination of the porosity of each layer separately and the calculation of the total porosity of the textiles, resulting from the porosity of their individual layers. Figure 7 shows the porosity results for the tested textiles. For each textile product, the total porosity *P*_t_, inner layer porosity *P*_in_, and outer layer porosity *P*_out_ were calculated. In the case of ILIAS, the porosity of the inner layer (microvelour backing) was calculated by taking into account the contribution of two adjacent thin layers: the middle upper (knitted fabric 1), and the middle lower (knitted fabric 2). The microtomography results show that the RIBCORD bright woven fabric is the most porous textile (*P*_t_ = 81%), while the ILIAS knitted fabric is the least porous (*P*_t_ = 55%). The same relationship applies to the porosity of the outer layer: *P*_out_ = 89% for RIBCORD bright and *P*_out_ = 74% for ILIAS. The observed correlation between total porosity and the porosity of the outer layer results from the significantly larger volume of these textiles in the outer layer compared to the other layers. On the other hand, RIBCORD bright has the least porous inner layer (*P*_in_ = 38%), while OREO has the most porous one (*P*_in_ = 56%).

Table 3 presents the parameters characterizing the yarns in the tested textiles. As can be seen in the RICORD bright and RIBCORD dark, micro-CT analysis of the fabrics forming the inner layer of these textiles revealed significant differences in porosity and yarn diameter depending on the warp and weft.

In the case of ILIAS, both knitted fabrics in the middle upper and middle lower layers differ significantly in density depending on the direction of the rows and columns. The observed significant anisotropy in the yarn structure and their weave parameters in knitted and woven fabrics may impact the functional parameters of the tested textiles.

### 3.2. Air Permeability of Textiles

**Figure 8:**
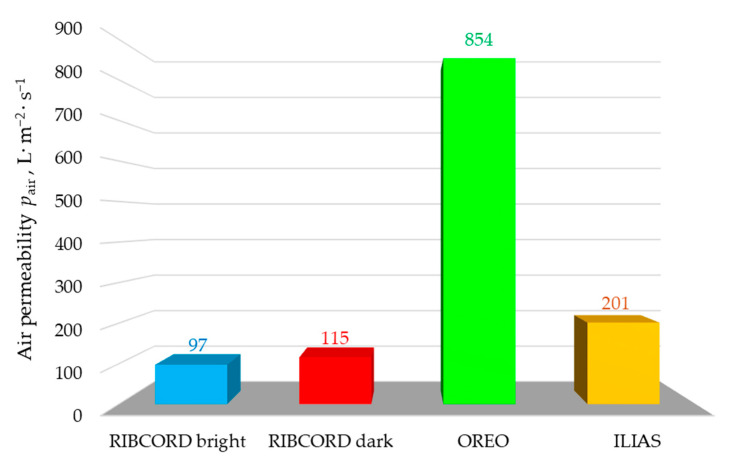
Air permeability of tested textiles.

Figure 8 presents the results of textile air permeability—*p*_air_ tests. The graph shows that the OREO has the highest *p*_air_ = 854 L·m^−2^·s^−1^. This woven fabric is characterized by very good breathability. This type of upholstery textile is well-suited for air-conditioned interiors or where high ventilation is required.

RIBCORD bright, RIBCORD dark, and ILIAS showed significantly lower *p*_air_ values: 97 L·m^−2^·s^−1^, 115 L·m^−2^·s^−1^, and 201 L·m^−2^·s^−1^. These types of upholstery textiles are well-suited for furniture in spaces where thermal comfort and resistance to drafts are essential. These may include offices or waiting rooms, for example. Analyzing the air permeability results shows a strong correlation with the porosity results of the inner textile layer (*P*_in_ in Figure 7). In each of the tested textile materials, the inner layer is the least porous and acts as the most effective barrier to airflow, which likely determines the air permeability of the entire textile product. It is important to realize that the porosity of a textile product is not the only parameter determining its air permeability. It is also influenced by the layer thickness and the spatial orientation of the fibers. These parameters determine the shape and size of the channels through which air flows.

### 3.3. Tensile Strength: Maximum Force and Elongation at Maximum Force of Textiles

**Figure 9:**
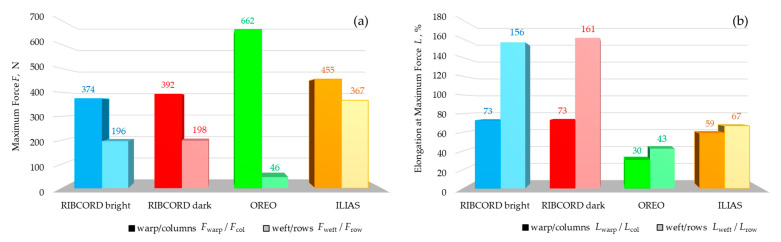
(**a**) Maximum force of tested textiles, (**b**) Elongation at maximum force of tested textiles.

The textiles were subjected to strength tests in accordance with EN ISO 13934-1:2013, which determined the maximum force (*F*) and elongation at maximum force (*L*). The test results are presented in Figure 9a,b.

Based on the results, RIBCORD bright and RIBCORD dark achieved very similar *F* and *L* values in both the weft (*F* = 196 N and *F* = 198 N; *L* = 156% and *L* = 161%, respectively), and warp directions (*F* = 374 N and *F* = 392 N; *L* = 73%, *L* = 73%, respectively), achieving category C (according to PN-EN 14465). The significant differences in *F* values depending on the stretching direction may be due to the larger yarn diameter in the warp than in the weft. OREO is characterized by the highest *F* value along the warp (662 N) and the lowest *F* value along the weft (46 N), as well as the lowest *L* values in both the weft (43%) and warp (30%) directions among all tested textiles. The high anisotropy of OREO’s strength may be due to the significant difference in yarn diameter (350 µm in the warp and 250 µm in the weft). Additionally, OREO is characterized by a thick fiber pile, which is obtained using a napping process. This involves mechanically pulling fibers from the fabric’s interior in one direction. Therefore, the yarns that make up the structure cannot be excessively twisted, which translates directly into reduced strength (in this case, in the weft direction). These *F* and *L* values allow OREO to achieve category A. For ILIAS, the following *F* values were observed: 455 N (in the column direction), 367 N (in the row direction), and the following *L* values: 59% (in the column direction), 67% (in the row direction). Comparable *F* and *L* values determined in both directions can be reflected in comparable values for yarn diameter, column density, and row density. Based on the obtained results, ILIAS achieves category B in accordance with PN-EN 14465.

### 3.4. Abrasion Test

**Figure 10:**
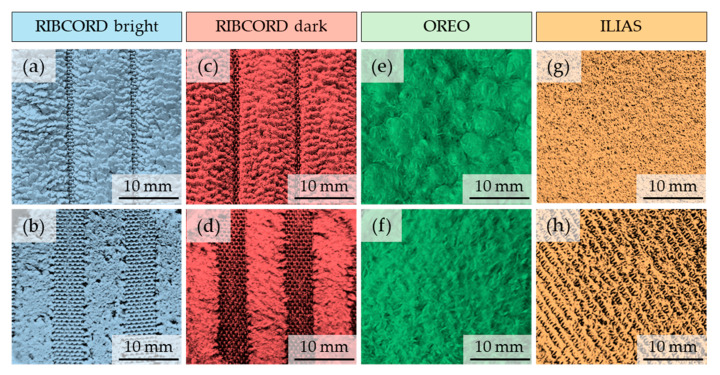
Photographs of the tested materials before (**a**,**c**,**e**,**g**) and after (**b**,**d**,**f**,**h**) the abrasion test.

Each of the four tested materials was used to prepare three working samples with a diameter of 38 mm. The study was conducted until 50,000 strokes were reached. The control range was set at 5000 strokes. The load was 795 g (12 kPa). According to the EN ISO 12947-2 standard, for pile materials, the criterion for destruction is “Fully worn off area”. Worn of area means area which has been denuded of pile or flock such that the ground fabric is exposed. If more than three-quarters of the surface of the casing is destroyed, the criterion of destruction is met. Figure 10 shows photographs of all tested materials before (a, c, e, g) and after (b, d, f, h) the abrasion test.

The test showed that all tested materials have high abrasion resistance. For RIBCORD bright, RIBCORD dark, and OREO woven materials, the destruction criterion was not met after 50,000 strokes of abrasion. For the ILIAS knitted fabric, the cover was wiped after 45,000 abrasion cycles. According to the PN-EN 14465, all tested materials meet the level of requirements for category A (≥45,000). For comparison, for category C, the level of requirements is between 10,000 and 20,000 abrasion cycles [37].

### 3.5. Pilling Resistance

Each of the four tested materials was used to prepare three working samples with a diameter of 140 mm. The tests were carried out in the ranges specified in the standard: 125, 500, 1000, 2000, 5000, and 7000 cycles. After each step, the level of pilling was checked according to a 5-point scale, according to which five means no changes and one means strong pilling occurring over the entire surface of the sample. In addition to pilling, the degree of fuzzing and matting of the fibers on the surface of the samples was also observed. For RIBCORD bright, RIBCORD dark, and OREO woven materials, after 2000 cycles, the degree of pilling reached level 4, and this value was maintained until the end of the study. According to the PN-EN 14465, this result was achieved at the level of requirements for category B. Among the tested materials for OREO fabric, a slight fuzzing of the fibers on the surface of the sample was recorded, which was sealed at level 4.

**Figure 11:**
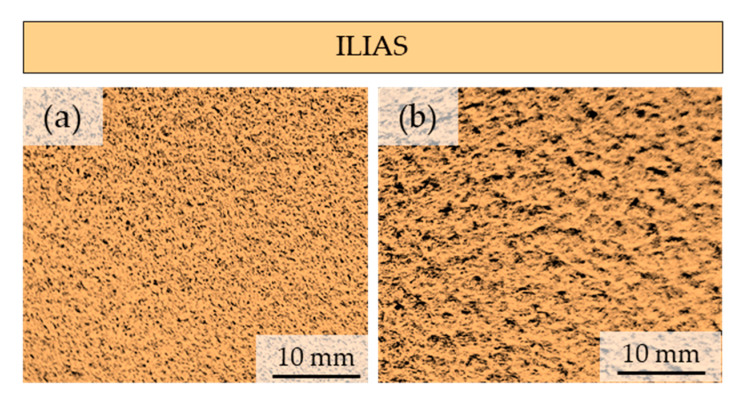
Photographs of the ILIAS knitted fabric: (**a**) before and (**b**) after the pilling test.

In contrast to the tested fabrics, the ILIAS knitted fabric sample showed very low resistance to pilling. After 2000 cycles, its level reached 2, and after 5000, it reached 1, which means “Strong pilling—piles of different dimensions and densities cover the entire part of the sample surface”. According to the PN-EN 14465 standard, such a result causes disqualification in terms of quality, as it does not fall into any of the categories from A to D. In addition, strong fuzzing of the surface was observed for ILIAS knitted fabric, as a result of which the phenomenon of matting of the fibers also occurred. Figure 11 shows a photograph of the ILIAS knitted fabric before and after the pilling test.

### 3.6. Flammability Assessment—Ignition Source Match Flame Equivalent of Textiles

The results of the flammability assessment of the tested textiles were presented in Table 4. The table contains observations regarding the course and results of the test and the textile classification assigned based on them.

The test process and results for RIBCORD bright and RIBCORD dark were identical: after 4–5 s of exposure to the ignition flame, the woven fabric ignited and the combustion process intensified, as a result of which the tested textile material had to be extinguished. The ILIAS test had a nearly identical course and result (ignition occurred after 5–6 s). The test with OREO had a completely different course: after 3–4 s of flame exposure, a hole appeared in the fabric, exposing the PU foam. Further contact of the ignition flame with the PU foam did not ignite it, and after the flame was removed, the combustion process spontaneously ceased. The following local damage to the system was observed: seat: 10 mm × 10 mm; backrest: 70 mm × 20 mm (shown in Figure 3). The OREO woven fabric, unlike the other three tested textiles, likely had a flame-retardant finish.

## 4. Conclusions

The aim of the research presented in this article was to evaluate the functional parameters of commercial upholstery textiles used in the furniture industry. Based on the tests, the textiles were classified in terms of user comfort and safety.

Three woven fabrics and one knitted fabric with a layered structure, differing in their spatial geometry, raw material composition, and surface mass, were tested. Statistical analysis of the textile microstructure was performed using high-resolution micro-CT. The comfort and safety of upholstery textiles were assessed based on measurements of air permeability, tensile strength, abrasion, and pilling resistance, as well as flammability. Based on the results obtained, the following conclusions can be drawn:

- The micro-CT analysis revealed differences in the fabrics’ fiber/yarn diameter *d*_FY_ and pore size *d*_P_. The RIBCORD bright and RIBCORD dark woven fabrics had similar average *d*_FY_ values (65 µm and 61 µm, respectively), while the ILIAS knitted fabric had the highest *d*_FY_ (73 µm).
- The micro-CT analysis revealed a difference in the textiles’ total porosity *P*_t_. The most porous fabrics were RIBCORD bright (*P*_t_ = 81%) and RIBCORD dark (77%), while the least porous fabric was ILIAS (*P*_t_ = 55%)
- A clear correlation was observed between air permeability and the porosity of the inner layer of the tested textiles. According to micro-CT analysis, the most air-permeable textile material was OREO fabric (*p*_air_ = 854 L·m^−2^·s^−1^) with the most porous inner layer (*P*_in_ = 56%).
- The anisotropy of the textiles in terms of their tensile strength was justified by microtomographic structural analysis of the yarn structure in the tested woven and knitted fabrics. Maximum force *F* and elongation at maximum force *L* strongly depend on the stretching direction. OREO woven fabric is the most resistant to tearing in the warp direction (*F*_warp_ = 662 N) and at the same time the least resistant in the weft direction (*F*_weft_ = 46 N) of all tested textiles. According to EN ISO 14465, the tested textiles were assigned the following categories: C (RIBCORD bright, RIBCORD dark), A (OREO), B (ILIAS).
- All tested materials showed very good abrasion resistance. In the case of pilling, the fabrics met an acceptable level of requirements, while the knitted fabric did not.
- Flammability tests have shown that the only flame-retardant textile is OREO woven fabric.

In summary, the tested upholstery textiles proposed by the same manufacturer are characterized by a significant diversity in their internal structure, which significantly impacts the various functional characteristics that influence their comfort and safety.

## Figures and Tables

**Table 1:** Parameters of the tested textiles.

Textile	Layer	Layer Type	Woven/Knitted Fabric Structure	Thickness [mm]		Composition	Mass Per Unit Area [g·m^−2^]
RIBCORD bright	outer	wide-wale corduor	−	2.10	2.70	88% polyester (PES), 12% polyamide (PA)	385
	inner	woven fabric	plain	0.60			
RIBCORD dark	outer	wide-wale corduor	−	2.10	2.70	88% polyester (PES), 12% polyamide (PA)	385
	inner	woven fabric	plain	0.60			
OREO	outer	irregular loop pile	−	2.50	3.10	100% polyester (PES)	470
	inner	woven fabric	plain	0.60			
ILIAS	outer	velour face	−	1.20	2.50	100% polyester (PES)	405
	middle upper	knitted fabric 1	single jersey	0.40			
	middle lower	knitted fabric 2	single jersey	0.30			
	inner	microvelour backing	−	0.60			

**Table 2:** Summary of research methods used.

Test Method	Device	Determined Parameters	Standard
X-ray microtomography	micro-CT SkyScan1272	fiber thickness porosity	not applicable
Air permeability measurement	air permeability tester: FX 3300	air permeability	EN ISO 9237
Tensile strength	Hounsfield H10KeS universal testing machine	maximum force and elongation at maximum force of textiles	EN ISO 13934-1
Abrasion and pilling resistance of fabrics by the Martindale	M235 Martindale	abrasion resistance pilling resistance	EN ISO 12947-2 EN ISO 12945-2
Ignition source match flame equivalent of textiles	flammability testing apparatus	flammability	EN 1021-2:2014

**Table 3:** Parameters of the textile yarn.

Textile	Layer	Layer Type	Yarn Porosity [%]		Yarn Diameter [µm]		Warp/Column Density [cm^−1^]	Weft/Row Density [cm^−1^]
			Warp/Column	Weft/Row	Warp/Column	Weft/Row		
RIBCORD bright	outer	wide-wale corduor	−	−	−	−	−	−
	inner	woven fabric	25	17	350	318	28	30
RIBCORD dark	outer	wide-wale corduor	−	−	−	−	−	−
	inner	woven fabric	26	17	350	318	28	30
OREO	outer	irregular loop pile	−	−	−	−	−	−
	inner	woven fabric	10	28	350	250	12	14
ILIAS	outer	velour face	−	−	−	−	−	−
	middle upper	knitted fabric 1	35	35	96	96	14	9
	middle lower	knitted fabric 2	27	27	162	162	17	25
	inner	microvelour backing	−	−	−	−	−	−

**Table 4:** Results of flammability testing of upholstery systems made with flame-retardant PU foam; EN 1021-2 (ignition source match flame equivalent of textiles).

Textile	Filling	Observed Phenomenon/Damage to the System	Evaluation
RIBCORD bright	Flame-retardant PU foam	After 4–5 s of exposure to the ignition flame, the system ignited—an intensifying burning process of the fabric and foam was observed. The system had to be extinguished.	Textile is flammable.
RIBCORD dark	Flame-retardant PU foam	After 4–5 s of exposure to the ignition flame, the system ignited—an intensifying burning process of the fabric and foam was observed. The system had to be extinguished.	Textile is flammable.
OREO	Flame-retardant PU foam	After 3–4 s of flame exposure, a hole melted in the fabric, exposing the PU foam. Contact of the ignition flame with the PU foam did not cause it to ignite. After the removal of the flame source, the burning process ceased spontaneously. Local damage to the system was observed as follows: seat: 10 mm × 10 mm; backrest: 70 mm × 20 mm.	Textile is self-extinguishing. Textile has a flame-retardant finish.
ILIAS	Flame-retardant PU foam	After 5–6 s of flame exposure, the system ignited—an intensifying burning process of the fabric and melting of the foam was observed. The system had to be extinguished.	Textiles are flammable.

## Data Availability

The original contributions presented in this study are included in the article. Further inquiries can be directed to the corresponding authors.
